# Relationship Between Cone-Beam CT Evaluation and Clinical Evaluation Before and After Orthodontic Treatment and the Rate of Gingival Recession: A Systematic Review

**DOI:** 10.7759/cureus.62536

**Published:** 2024-06-17

**Authors:** Lujain Alsulaimani, Mohammad Qali

**Affiliations:** 1 Department of General Dentistry, Al Baha Specialized Dental Hospital, Ministry of Health, Al Bahah, SAU; 2 Department of Surgical Sciences, College of Dentistry, Health Sciences Center, Kuwait University, Kuwait, KWT

**Keywords:** digital, systematic review, gingival recession, orthodontic treatment, cbct

## Abstract

A systematic review was conducted to investigate the correlation between gingival recession and orthodontic treatment analysis using cone-beam CT (CBCT) dental images to provide an accurate overview of the factors that can influence gingival recession. The literature was searched across several databases (PubMed, Scopus, Web of Science, etc.) for studies using CBCT dental images. Four full-text articles describe how CBCT is used to evaluate gingival recession during orthodontic treatment. The Risk-of-Bias VISualization tool was used to assess the risk of bias. In dentistry, CBCTs are used for various imaging modalities. An accurate assessment of gingival recession was made using CBCT. There were only 35 (22.5%) cases of retraction of the gingival margin after orthodontic treatment. In most studies, various types of malocclusions were treated successfully with fixed orthodontic treatment without gingival recession. Among the studies, 50% had a low risk of bias in all four areas, while one study (25%) had an unclear risk of bias. Only one study (25%) had a high risk of bias. CBCT systems have been extensively studied to show their wide application potential in preventing gingival recession. CBCT systems should be further investigated to address limitations associated with methodology and application. Dental applications of CBCT can be enriched by overcoming these challenges.

## Introduction and background

The recession of the gingiva results from an apical shift of the gingival margin concerning the cementoenamel junction (CEJ) where the root surface is exposed [1]. Gingival recession can expose the roots of the teeth, resulting in tooth hypersensitivity and root caries, which can impair conservative and prosthetic treatment options [2]. In one study, there was at least one recession in one site for 50% of people aged 18-24 years and 88% of people aged 65 or older [3]. Gingival recession typically occurs in the mandibular incisors and maxillary molars [3].

Several factors may contribute to gingival recession following orthodontic treatment. These factors include skeletal aspects, such as thin cortical bone covering the tooth root, potentially leading to dehiscence or fenestrations; gingival considerations, such as a thin gingival phenotype, improper frenulum attachment, shallow oral vestibule, or elevated mental muscle attachment; and dental elements, including the alignment of vestibular teeth, overcrowding, rotation, and abnormal tooth eruption [4,5]. Literature on orthodontics and periodontology frequently discusses the association between orthodontic treatment and pathology in the periodontium. In some studies, orthodontic treatment has been linked to gingival recession, and some studies have found that gingival recession deepens following orthodontic treatment [6,7] In contrast, other studies have not demonstrated an evolution of gingival recession after orthodontic treatment [8,9]. In the absence of adequate alveolar bone support, apical migration can occur, eventually leading to gingival recession [10].

It has been observed that patients who have had orthodontic treatment may experience more gingival recession if the margin of their free gingiva is less than 0.5 mm thick, particularly if their anterior teeth are tilted forward post-treatment [11]. Orthodontic teeth movement is believed to occur through resorption and bone formation in the areas of the periodontal ligament where pressure or tension is applied. This occurs when force systems result in extreme compression of the periodontal ligaments and hyalinization of the adjacent bone tissues. When the hyalinized tissue is removed by undermining resorption, the alveolus is widened, and the tooth is loosened [11]. The findings of Wennstrom et al. [10] demonstrate that maintaining a tooth position beyond that limit results in the loss of bone support (dehiscence), which is a risk factor for gingival recessions. When a tooth is properly positioned within the alveolar bone, it stimulates bone formation at about 50% of its height while maintaining the health of the gingiva. An appropriate amount of force should be applied to move the tooth effectively, which is enough to stimulate the tooth’s movement but gentle enough to avoid causing harm to the surrounding tissue [12].

Gingiva normally lies within 0.5 to 2.0 mm of the CEJ in a healthy periodontium. A gingival recession occurs when it moves beyond the CEJ [13]. A dental probe is used to clinically measure gingival recession from the crest to the CEJ. Plaster models are useful for assessing gingival recession in patients where intraoral measurement is not feasible. They provide a three-dimensional (3D) view that allows for a detailed evaluation of impressions obtained during a clinical examination without interfering with the soft tissues within the mouth [14]. However, study casts have disadvantages, such as physical and chemical damage, wear and tear, and distortion. Additionally, plaster models are not cost-effective or time-efficient [15]. Despite multiple methods for assessing periodontal biotypes, their accuracy remains uncertain [16,17].

In recent years, digital methods have been developed to measure periodontal biotypes through scanning and assessment. Digital model scanning and cone-beam CT (CBCT) are commonly used in orthodontic treatment for diagnostic and evaluation purposes. Digital models offer several advantages, including efficient handling and storage of data, time-saving, and reduced errors from electronic data transfer and storage [18,19]. Using these non-invasive and more accurate methods has been proven effective for measuring soft and hard tissues, as well as changes in gingival margin position and gingival thickness (GT) [20].

There have been limited investigations into the link between gingival recession before and/or after orthodontic treatment and the initial and final conditions of periodontal tissues using the CBCT system. As a result, we conducted an extensive review to explore the impact of orthodontic treatment on the site of periodontal tissues and the level of gingival recession using the CBCT system. Moreover, we analyzed various factors that may influence gingival recession during orthodontic treatment.

## Review

Methodology

Study Protocol and Research Question

Two independent reviewers conducted a systematic review using Preferred Reporting Items for Systematic Reviews and Meta-Analyses (PRISMA) criteria on the relationship between CBCT evaluation and clinical evaluation before and after orthodontic treatment, as well as the rate of gingival recession. The research protocol was enlisted with PROSPERO, the International Prospective Register of Systematic Reviews (registration number: CRD42023481004).

The following research question was explored in this systematic review: what are the effects of orthodontic appliances on gingival health in patients before receiving orthodontic therapy compared to patients after orthodontic therapy with/without gingival recessions? The PICO is presented in Table 1.

**Table 1:** A detailed description of the PICO elements.

PICO question	What are the effects of orthodontic appliances on gingival health with patients before receiving orthodontic therapy compared to patients after orthodontic therapy with/without gingival recessions?
Population (P)	Patients with CBCT scans who were exposed to orthodontic therapy
Intervention (I)	Orthodontic therapy before receiving treatment with/without gingival recession
Control (C)	Patients after orthodontic therapy with/without gingival recession
Outcome (O)	Excellent orthodontic therapy in patients with/without gingival recession

The CBCT scan records the population exposed to orthodontic therapy (P), before orthodontic therapy without gingival recession (I), after orthodontic therapy with/without gingival recession (C), whether there is an association between gingival recession and orthodontic therapy (O), and human interventional studies (randomized and non-randomized(s)).

Selection Criteria

Inclusion criteria: The articles needed to be in vivo studies conducted on male and female adult subjects over 18 years of age with good physical health. The manuscript needed to be in English with full-text access, and the patient must not have had gingival recession or restorations affecting the CEJ. The use of good-quality X-rays was required to allow for the identification of landmarks and control of head rotation. Additionally, CBCT records before and after orthodontic treatment were required. A limited number of papers were accepted that reported on human interventional studies (randomized and non-randomized). Finally, this systematic review was constrained by a timeline between 2010 and 2023 (Table 2).

**Table 2:** A detailed description of the inclusion and exclusion criteria. CBCT = cone-beam computed tomography; CEJ = cementoenamel junction

Inclusion criteria	Exclusion criteria
In vivo studies (on humans)	In vitro studies (on animals)
Male/Female human adult patients in good physical health over 18 years old	Studies that included prior periodontal surgery, orthodontic treatment, etc.
Written in English with full-text access	Published in any language other than English
A condition without gingival recession or restorations involving the CEJ	Previous dental trauma, drug-induced gingival enlargement, periodontitis of teeth, missing teeth, and deformation resulting from trauma
Good quality X-rays enabling the identification of landmarks and control of head rotation	With an irrelevant outcome about gingival recession/orthodontic treatment
Availability of CBCT records before and/or after orthodontic treatment	Measuring the gingival recession before and/or after orthodontic treatment with something other than CBCT
Restricted to human interventional studies (both randomized and non-randomized)	Studies published in journals not cited in the open-access checklist for predatory publishers
Time constraints between 2010 and 2023	With an irrelevant outcome about gingival recession/orthodontic treatment

Exclusion criteria: All non-English-language studies, as well as in vitro (animal) studies, were excluded. Studies including patients who had prior periodontal surgery, orthodontic treatment, dental trauma, or drug-induced gingivitis; studies published in a journal not cited in the open-access checklist for predatory publishers, periodontitis of teeth, missing teeth, deformations caused by trauma; studies in which gingival recession was measured before and/or after orthodontic treatment by a method other than CBCT; and studies with an outcome unrelated to gingival recession/orthodontic treatment were excluded. Study participants needed to have met the inclusion criteria and undergone successful treatment, regardless of whether successful treatment was an inclusion criterion (Table 2).

Critical Appraisal and Search Strategy

Following PRISMA guidelines, two separate reviewers assessed the suitability of the titles and abstracts of the studies. In the event of any disagreement, a senior reviewer provided consultation. There was a discussion between the reviewers to resolve any discrepancies.

A comprehensive search was conducted in December 2023. We searched the following electronic databases for literature on this topic: PubMed, Scopus, Web of Science, and Google Scholar digital databases. The following list of keywords and Boolean operators were used in the search (“AND,” “OR”): [(gingival recession) OR (gum recession) OR (receding gums)] AND [(orthodontic treatment) OR (orthodontic therapy) OR (conventional orthodontic)] AND [(CBCT system) OR (CBCT scan) OR (digital scan)] AND [(tooth angulation) OR (tooth movement)] AND [(bone level) OR (periodontium)]. After conducting an electronic search, we manually searched the reference lists of the included articles. Additionally, we contacted some authors for further information.

When conducting the search in December 2023, no recent reviews were available, so we conducted an electronic search for relevant articles published in English between 2010 and 2023. Unfortunately, very few articles and reviews related to this research topic are available in the dental field.

Data Extraction

Two reviewers carefully extracted all relevant information from each study included in the analysis. The collected data were then entered into a pre-designed Microsoft Excel (Microsoft Corp., Redmond, WA, USA) collection form created specifically for this purpose. The following information was collected and organized into columns: author and year of the study, sample size, state of the gingiva before and after orthodontic treatments, types of orthodontic treatments, and clinical results regarding the gingival recession rate. The findings were then analyzed descriptively as supplemental data.

Risk of Bias Assessment

Approved studies were evaluated for bias using the Risk-of-Bias VISualization (Robvis) [21] tool. Two independent reviewers completed and verified the assessment. The risk of bias assessment tool (Robvis tool) included the following seven specific domains: random sequence generation, allocation concealment, blinding of participants and personnel, blinding of outcome assessment, incomplete outcome data, selective reporting, and other biases. The domains were classified as “high,” “unclear,” or “low” based on the results. Every study selected was reported with its risk of bias values. The authors classified each selected study’s risk of bias according to (1) there was a low risk of bias in each domain; (2) bias risk was rated as “unclear” in at least one domain; and (3) at least one domain had a high bias risk rating.

Outcomes Measurements

Primary outcomes: Studies have demonstrated an effective method for evaluating and measuring the rate of gingival recession using CBCT systems for clinical evaluations before and after orthodontic treatment.

Secondary outcomes: The results of our study demonstrated that a variety of factors may have an impact on gingival recession during orthodontic treatment.

Tables were used to organize the data items. The following data items were prepared in one table: author and design, year of the study, sample, gender, age of patients, types of teeth, gingiva state before and after orthodontic treatments, orthodontic treatment types, and clinical results regarding the gingival recession rate. The results were synthesized descriptively as complementary data.

Statistical Analysis

Due to the heterogeneity of the articles selected, no meta-analysis was performed. As a result, the outcomes were mainly evaluated descriptively.

Results

Study Selection

**Figure 1:**
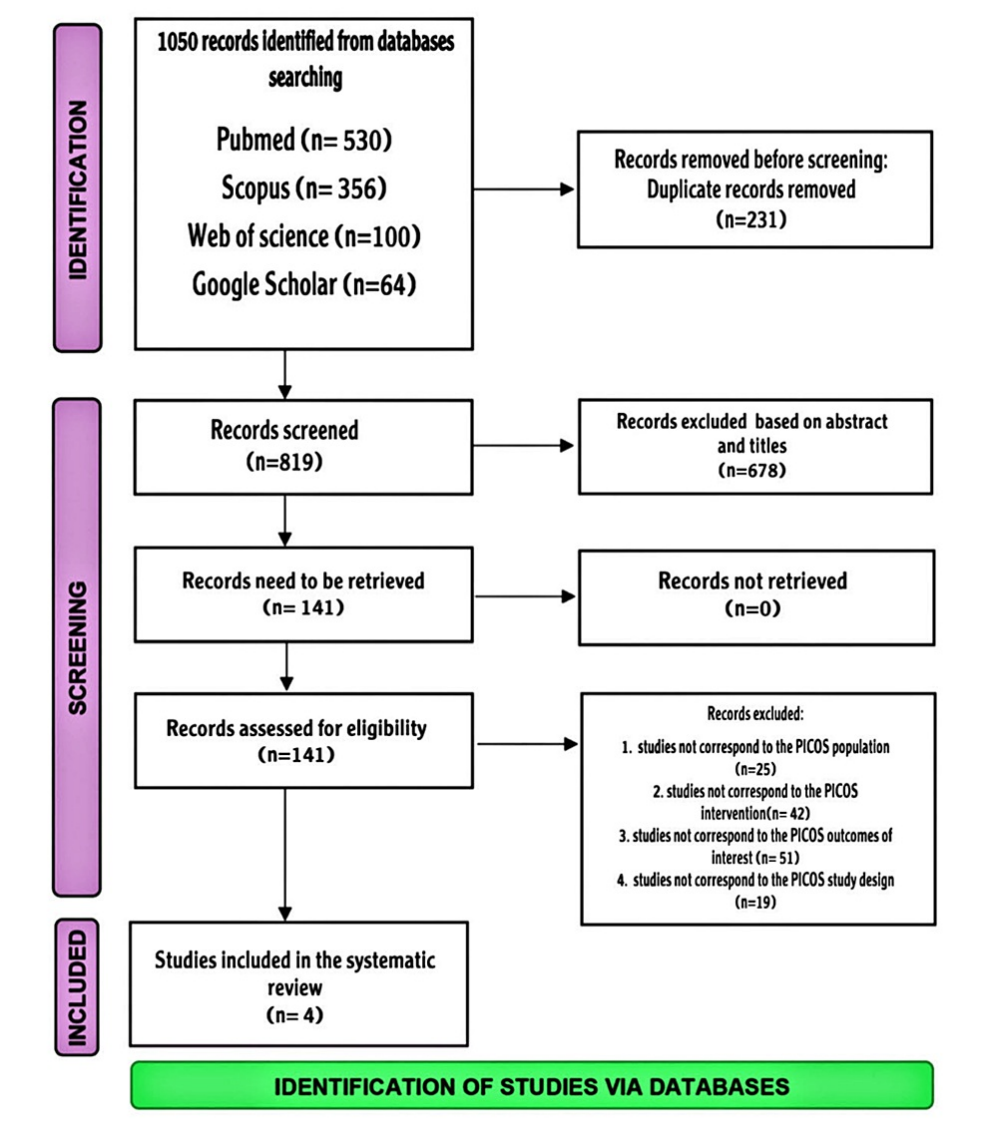
Preferred Reporting Items for Systematic Reviews and Meta-Analyses flow diagram illustrating the study selection process.

In the initial review of the titles and abstracts, 1,050 records were found in all databases. The remaining 819 articles were screened after removing the duplicate records. We excluded 678 articles based on abstracts and titles. The eligibility of 141 full-text articles was verified after screening and selection. Finally, 133 articles were excluded for various reasons. The systematic review ultimately included only four studies (Figure 1).

Study Characteristics

Four human studies were selected for review within the past 13 years. All of these studies met the eligibility criteria. None of the studies met any of the exclusion criteria. Researchers examined the development of gingival recession in patients with fixed orthodontic appliances. The CBCT system provided accurate assessments of the probability of developing gingival recession. This systematic review included the following types of studies: one prospective study [22] and three retrospective studies [23-25]. The four studies involved a total of 159 patients and 972 teeth. Study participants ranged from 24 to 408, with ages ranging between 16 and 53 years. The studies were reported from Poland [22], Korea [23,24], and Brazil [25]. The gender of the patients was reported in four studies (60 male patients and 112 female patients) [22-25] A total of 972 teeth were present, of which 948 were anterior teeth, while no posterior teeth were present [22-25]. A general focus of the three studies was the growth of gingival recession before and after orthodontic treatment [23-25], and one study examined the progress of gingival recession following orthodontic treatment [22]. According to the included studies in this review, CBCT scans were mainly used to assess soft tissue dimension concerning the CEJ in 30 to 52 cases, and the total number of cases was 157. In this study, 157 cases were collected in the following manner: 30 cases were extracted from prospective studies [22], and 117 cases were extracted from retrospective studies [23-25]. In total, 30 cases were examined to determine whether gingival recession develops after orthodontic treatment [22]. We studied 127 cases primarily to determine the rate at which gingival recession developed before and after orthodontic treatment [23-25] (Table 3).

**Table 3:** A comprehensive summary of the characteristics of the included studies.

Author/Year of the study	Study design	Sample	Gender	Age of patients (mean ± SD)	Type of teeth	Orthodontic treatment types	Gingiva state before orthodontic treatments	Gingiva state after orthodontic treatments	Clinical main outcomes regarding the gingival recession rate
Kalina et al., 2020 [22]	Prospective study	32 adults	Males: 14; females: 18	Age: 25.08 ± 6.50 years	180 mandibular teeth, including 120 incisors and 60 canines	Fixed orthodontic appliances	Evaluation not included in the study	15 teeth spontaneous complete improvement of pre-existing gingival recession, on two incisors, gingival recession decreased, and on three teeth, gingival recession did not change, while three teeth developed new defects	Properly planned changes in lower incisor and canine inclination can be carried out in adult patients without posing a high risk to labial gingival recessions if the individual periodontal biotype is respected
Lee et al. (2019) [23]	Retrospective study	45 adults	Males: 10; females: 35	Age: 21.58 ± 8.82 years	360 anterior teeth including 180 central incisors and 180 lateral incisors	Fixed orthodontic appliances	The mean distance from the cementoenamel junction to the gingival margin was 1.69 ± 1.01 mm before orthodontic treatment	The mean distance from the cementoenamel junction to the gingival margin was 1.55 ± 1.01 mm after orthodontic treatment. The mean amount of gingival margin change (i.e., gingival recession) was 0.14 ± 0.57 mm	Gingival recession tends to develop in association with the proclination of a tooth, rather than retroclination. As the inclination of a tooth increased labially, gingival recession increased by approximately 0.2 mm per 1°
Kim et al. (2018) [24]	Retrospective study	52 adults	Males: 19.6%; females: 80.4%	Mean age: 22 years	408 teeth including 208 central incisors and 200 lateral incisors	Fixed orthodontic appliances	There was a significant correlation between gingival thickness and alveolar bone thickness, which was evident only before orthodontic treatment	Gingival thickness was not statistically associated with tooth inclination or rotation. Similarly, alveolar bone thickness was also not statistically associated with tooth inclination (neither proclination nor retroclination); however, it was significantly associated with tooth rotation. Specifically, greater tooth rotation was associated with a greater reduction in alveolar bone thickness	Gingival thickness and alveolar bone thickness can be accurately assessed by comparing sectioned CBCT images and virtual models
Castro et al. (2016) [25]	Retrospective study	30 adults	Males: 11; females: 19	Mean age: 13.3 years	24 teeth including 60 buccal and 60 lingual surfaces for each, maxillary and mandibular teeth	Fixed orthodontic appliances	The distance from the cementoenamel junction to the bone crest was greater than 2 mm in 162 (11%) of the 1,440 root surfaces before orthodontic treatment	The distance from the cementoenamel junction to the bone crest increased to 19% in 270 after orthodontic treatment	The distance from the cementoenamel junction to the bone crest changed and increased after orthodontic treatment

Primary Outcomes

There were 120 successful cases without gingival recession across the four studies that used CBCT as the primary 3D radiographic measurement (77.4%). A total of 35 (22.5%) cases among the 155 orthodontic cases included in all four studies had a gingival recession [22-25]. Of the 155 fixed orthodontic treatment cases, 120 (77.4%) cases had a high success rate in treating different types of malocclusions without gingival recession. There were only 35 (22.5%) cases of retraction of the gingival margin after orthodontic treatment, most commonly resulting from proclination rather than retroclination of a tooth. Most of these studies showed a high success rate for fixed orthodontic treatment in treating various types of malocclusions without gingival recession: 73.3% (22 cases) in the study by Kalina et al. [22], 82.2% (37 cases) in the study by Lee et al. [23], 90% (50 cases) in the study by Kim et al. [24], and 85.1% (30 cases) in the study by Castro et al. [25].

Secondary Outcomes

Three studies reported factors that may influence gingival recession during orthodontic treatment as secondary outcomes [22-24]. These studies examined factors associated with gingival margin changes, which were categorized into the following four categories: patient characteristics (e.g., age and gender), tooth characteristics (e.g., jaw and position), periodontal characteristics (e.g., GT and ABT), and orthodontic characteristics (e.g., history of orthognathic surgery, tooth inclination, and tooth rotation). Both studies found a non-significant association between alveolar bone thickness and labial GT with gingival recession following orthodontic treatment. Furthermore, after considering age, sex, the position of the tooth in the jaw, the history of orthognathic surgery, and the degree of rotation and angulation of the teeth, the only variable that showed statistically significant differences was the angulation of the teeth [22,23]. One study found that patients who underwent orthognathic surgery had a statistically significant change in GT. ABT did not significantly differ between individuals with orthodontic treatment versus those without based on sex, tooth arch, tooth position, or orthognathic surgery [24].

Risk of Bias Assessment

**Figure 2:**
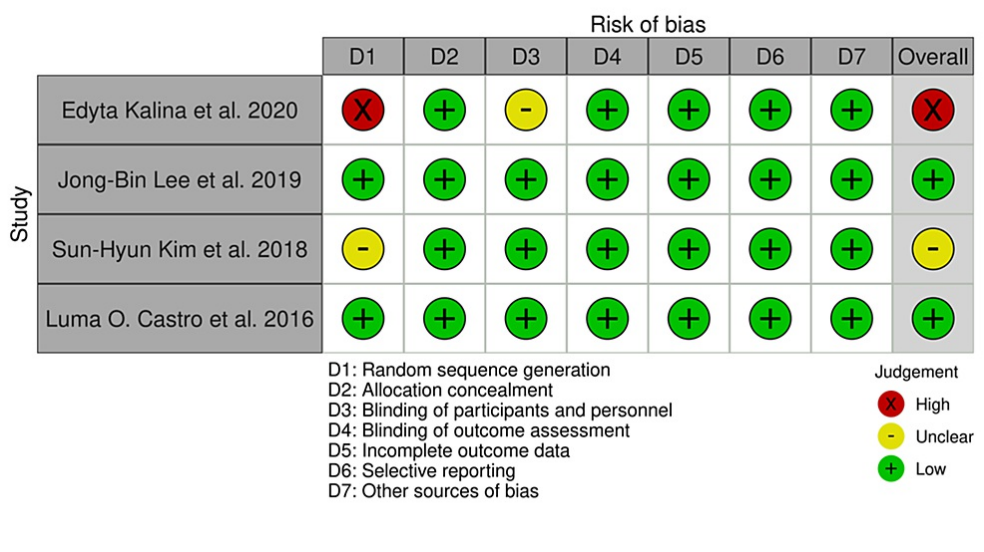
Risk of bias summary of the included studies.

**Figure 3:**
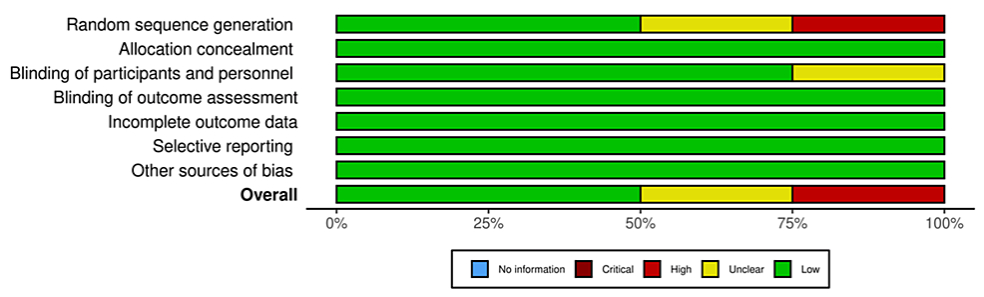
Risk of bias graph of the included studies.

According to this review, most studies in the following categories had a low risk of bias: random sequence generator (50%), incomplete outcome data (75%), and selective reporting (75%). Allocation concealment, blinding of participants and personnel, blinding of outcomes assessment, and other sources of bias were also found to have a low risk of bias (100%), as shown in Figure 2. We found two studies (50%) that had a low risk of bias [23,25]. Among 25% of studies, there was an unclear risk of bias [24]. In one study, the risk of bias was high (25%) [22]. One study had an unclear risk of bias in certain domains due to insufficient information to make a definitive determination [24]. Another study had a high risk of bias due to a lack of randomization, as shown in Figure 3 [22].

Discussion

Few studies in the literature have explored the connection between gingival recession and fixed orthodontic treatment using digital measurements (CBCT). Therefore, we conducted a systematic review to examine this relationship using the CBCT system. This is important because orthodontic treatment is becoming increasingly popular among children, adolescents, and adults [26]. Alveolar bone remodeling around teeth and periodontal tissues in orthodontics may be influenced by various biological events, such as GT, proclination, treatment duration, and type of treatment [27]. A significant correlation has been found in most studies [28-30] between the proclination of a tooth and gingival recession during orthodontic treatment. Several periodontal parameters must be analyzed quantitatively and qualitatively to plan periodontal treatment [16,31,32]. Furthermore, this can be used in orthodontics [33], implant therapy [34-36], and conventional prosthodontics [37,38] when a change in the inclination of teeth is expected.

The periodontal tissue dimensions must be accurately assessed before orthodontic treatment begins to achieve the best aesthetic result. To determine gingival biotypes (i.e., GT), several methods have been proposed [16]. In clinical practice, probe transparency and visual inspection are easy to use and time-efficient, but their accuracy is controversial [39]. Additionally, transgingival probing is another straightforward and commonly used method that measures GT directly using a periodontal probe to pierce the gingiva directly and assess its thickness [40]. The disadvantages of this method include the invasiveness and risk of errors associated with the angulation of the probe during penetration, as well as the requirement of local anesthesia that causes discomfort to the patient [41,17,42]. Optical coherence tomography is a non-invasive, comfortable imaging technique that uses light to create cross-sectional images of gingival tissues that produce high-resolution images [43,44]. The cost of the equipment and the requirement for trained personnel may limit the accessibility of this method [43,44].

Although ultrasound can be a comparatively good option as it is non-invasive, determining the location for accurate measurements is challenging. To address patient pain and resistance to orthodontic diagnosis and periodontal treatment, unlike traditional methods that may require physical probing and contact, in recent years, researchers have focused on developing non-invasive techniques for measuring gingival tissue using CBCT [42,45]. This significantly reduces patient discomfort and facilitates smoother diagnostic procedures as well as improving patient compliance [45]. Furthermore, CBCT has been recommended as a gold-standard method for determining gingival health in a few studies, and with the ability to provide high-resolution 3D images, allowing for precise measurement of gingival tissue. This accuracy is critical for effective diagnosis and treatment planning in orthodontics and periodontics [46,47]. The 3D imaging capability of CBCT enables clinicians to visualize bone thickness without overlapping between the buccal and lingual cortical bones and teeth, thereby enabling orthodontic movement to be measured as a rise in the gap between the CEJ and the crest of the alveolar bone [16,23,48-55]. Moreover, as suggested by Cesur et al. [56], as technology has advanced, CBCT devices emit lower levels of radiation, making them applicable to a wide variety of dental procedures. On the other hand, Kloukos et al. [57] showed zero difference between the GT measurements taken by ultrasound and CBCT. A range of 0.13 mm to 0.21 mm difference was observed when CBCT measurements were compared with ultrasound measurements, especially at a marginal level.

Different opinions exist concerning the effects of orthodontic treatment on gingival recession [8,9,58,59]. An individual may experience gingival recession for various reasons, and one or more of these factors usually lead to its appearance [60]. Some previous studies have reported that tooth movement after orthodontic treatment is one of the major causes of gingival recession, as are gingival margins of at least 0.5 mm, dehiscence or thin alveolar bone, proclination, and orthognathic surgery [8,9,53,54]. Some studies have observed the association between keratinized tissue width (KTW) and GT concerning periodontal factors [4,6,61-67]. Gingival recessions occur with a frequency of 20% in patients without periodontal maintenance at sites with little or no attached gingiva. However, there was no further recession in teeth with large zones of attached gingiva. Due to their proneness to gingival recession, the central and lateral incisors were the only teeth examined in the four studies.

However, two studies [23,24] demonstrated that GT during orthodontic treatment is not associated with gingival recession. In one study [22], the data indicated that GT decreased significantly, although KTW did not change significantly. According to orthodontic factors, there is an association between buccolingual GT and tooth inclination. According to the findings of the presented studies, post-orthodontic tooth inclination increases gingival recession [22-25]. Our study found that gingival recession on the labial segment was more likely to result from proclination than retroclination, where if the proclination of a tooth increased by 1°, then gingival recession would increase by approximately 0.2 mm. According to some speculation, a tooth’s root approaches the cortical bone because of its proclination. In turn, this may result in bone thinning or dehiscence formation, which may then result in gingival recession [68]. When considering different therapeutic options for adult patients, controlled proclination provides a noteworthy alternative to extraction. Although numerous studies have reported an association between tooth proclination and gingival recession, according to Laursen et al., after analyzing 12 cases of adult orthodontic therapy, the average depth, width, and area of recession decreased by 23%, 38%, and 63%, respectively, after orthodontic correction due to the root centering effect within bone housing [69].

According to Antanavičienė et al., 58.7% of recession cases improved with orthodontic treatment, 36% remained stable, and only 5.3% progressed [70]. Again, the accessibility of CBCT data could contribute to examining the changes in the proclination of tooth roots and in what manner they are linked to the margins of the bone envelope. However, Antonarakis et al. [71] reported that patients with greater incisor proclination were more likely to have multiple gingival recessions after treatment. Additionally, gingival recession and bone loss are more common among patients with skeletal class III mandibular incisors than in maxillary [72], and it was found that open-bite conditions impeded the improvement of gingival recession after orthodontic treatment [70]. Ji et al. found that orthodontic treatment in patients with open bites and infraversions significantly increased the incidence of gingival recession post-treatment [73]. It has been suggested that people who undergo orthognathic surgery are at a greater risk of losing alveolar bone due to scars forming from postoperative soft tissue contractions [74]. Despite this, most studies did not demonstrate a correlation between gingival recession and other factors, such as age, gender, jaw position, or previous orthodontic surgery [22,23,25]. Additionally, previous studies suggest that gingival recession following orthodontic treatment has a low prevalence and mild severity [4,5,75]. Furthermore, only one study demonstrated that gingival tissue was reduced in patients undergoing orthognathic surgery. This discrepancy may result from a different measurement method [24].

Limitations of the study

First, insufficient studies have examined the connection between orthodontic treatment and gingival recession using digital measurement (CBCT), and recession sites were relatively few. This limitation significantly affects the overall robustness of our findings. Data scarcity can introduce bias, as the limited sample size may not adequately represent the general population. Additionally, the small number of recession sites analyzed may not capture the full variability of outcomes, potentially skewing the results. Consequently, these factors limit the generalizability of our findings, emphasizing the need for further research with larger sample sizes to obtain more reliable and comprehensive results. As a result of the retrospective nature of most of the studies, several factors that could influence gingival recession were not entirely accounted for. Finally, gingival recessions may occur several years following debonding, posing another important limitation. Nevertheless, follow-up on the investigated population for an extended period is intended. To determine whether orthodontic tooth movement is related to gingival recession, a larger sample of well-controlled prospective studies or randomized controlled trials with a longer observation period of patients from various medical centers should be reported. To understand the presented outcomes and identify other predictors of gingival recession, a clinical examination should include an assessment of the gingival phenotype and oral hygiene. Furthermore, in future related studies, the study methodology should be improved by using a multidisciplinary approach involving orthodontists and periodontists performing CBCTs for periodontal tissue assessment and root position assessment.

## Conclusions

Based on the results of this systematic review and the literature, to avoid a high risk of gingival recession, orthodontic correction of tooth position (angulation, inclination) should be meticulously planned in adult patients. Considering the orthodontic principles that maintain the roots of the teeth inside the alveolar bone, it is essential to accurately assess gingival recession risk in orthodontic patients and develop a treatment plan during diagnosis. Orthodontists must exercise caution and careful consideration when opting to procline incisors, and it is recommended that they collaborate with a periodontist before and after their orthodontic assessment of periodontal tissues. By combining the specialized knowledge and clinical expertise of both disciplines, the study methodology, data interpretation, and, ultimately, the clinical recommendations can be substantially improved. Periodontists can provide vital insights into baseline periodontal conditions, potential risk factors, and expected responses to orthodontic movements. This can help orthodontists make more informed decisions about treatment planning and execution.
